# Clinical and Genetic Characterization of Noonan Syndrome in a Romanian Cohort from Transylvania: Details on *PTPN11* c.922A>G Variant and Phenotypic Spectrum

**DOI:** 10.3390/diagnostics15212753

**Published:** 2025-10-30

**Authors:** Florina Victoria Nazarie, Diana Miclea, Crina Șufană, Alina Botezatu, Radu Anghel Popp, Ionela Maria Pascanu, Camelia Alkhzouz, Simona Bucerzan, Călin Lazăr, Cecilia Lazea, Romana Vulturar

**Affiliations:** 1 Discipline of Medical Genetics, “Iuliu Hațieganu” University of Medicine and Pharmacy, Cluj-Napoca, 6, Pasteur St., 400349 Cluj-Napoca, Romania; nazarie.florina.victoria@elearn.umfcluj.ro (F.V.N.); anghel.popp@umfcluj.ro (R.A.P.); 2 1st Pediatric Discipline, Mother and Child Department, “Iuliu Hațieganu” University of Medicine and Pharmacy, 68, Calea Moților St., 400370 Cluj-Napoca, Romania; calkhuzouz@umfcluj.ro (C.A.); sbucerzan@umfcluj.ro (S.B.); calin.lazar@umfcluj.ro (C.L.); cecilialazea@umfcluj.ro (C.L.); 3 Medical Genetics Compartment, Children’s Emergency Clinical Hospital, 400370 Cluj-Napoca, Romania; 4 1st Pediatrics Clinic, Children’s Emergency Clinical Hospital, 68, Calea Moților St., 400370 Cluj-Napoca, Romania; crina@sufana.ro; 5 Faculty of Medicine, “Iuliu Hațieganu” University of Medicine and Pharmacy, 34, Victor Babes St., 400012 Cluj-Napoca, Romania; botezatu.alina@elearn.umfcluj.ro; 6 Department of Endocrinology, George Emil Palade University of Medicine, Pharmacy, Sciences and Technology of Târgu Mureș, 46, Gheorghe Marinescu St., 540136 Târgu Mureș, Romania; ionela.pascanu@umfst.ro; 7 Discipline of Cell and Molecular Biology, “Iuliu Hațieganu” University of Medicine and Pharmacy, Cluj-Napoca, 6, Pasteur St., 400349 Cluj-Napoca, Romania; romanavulturar@gmail.com

**Keywords:** Noonan syndrome, RAS/MAPK pathway, RASopathies, *PTPN11* c.922A>G (p.Asn308Asp), pulmonary valve stenosis, Williams–Beuren syndrome, neurofibromatosis–Noonan syndrome

## Abstract

**Background**: Noonan syndrome (NS) is a genetically heterogeneous condition within the RASopathies spectrum, with distinctive craniofacial features, congenital heart defects, short stature, and variably present developmental delay. Most cases result from variants in genes regulating the RAS/MAPK pathway, with *PTPN11* variants being the most frequent; the c.922A>G substitution being among the most commonly reported. **Methods**: This pilot study analyzed clinical and partial genetic features of NS in a cohort from Transylvania, evaluated in the Children’s Emergency Clinical Hospital in Cluj-Napoca. Thirty-one patients fulfilling the Van der Burgt diagnostic criteria (twenty-two males, nine females) were included. Clinical data were systematically reviewed, and targeted molecular testing for the *PTPN11* c.922A>G variant was performed. **Results**: Congenital heart defects were highly prevalent, with pulmonary stenosis representing the most frequent anomaly (54.8%). Craniofacial dysmorphism was observed in 76.7% of cases, cryptorchidism in 50% of the males, and short stature below the third percentile was described in 77.4% of patients. Genetic screening identified the *PTPN11* c.922A>G variant in two individuals (6.45%). Additional diagnoses included Williams–Beuren syndrome and a 17q11.2 deletion consistent with Neurofibromatosis–Noonan syndrome, underscoring the clinical and genetic heterogeneity of the cohort. Comparison with international reports highlighted variability in phenotype and variant frequency. Future research directions include Sanger sequencing of key *PTPN11* exons and the application of next-generation sequencing targeting all RAS pathway genes. **Conclusions**: This is the first Romanian cohort study on patients with a clinical suspicion of NS, providing insight into their evaluation. The findings reinforce the need for comprehensive molecular approaches, facilitating diagnostic precision and counseling strategies.

## 1. Introduction

**Figure 1:**
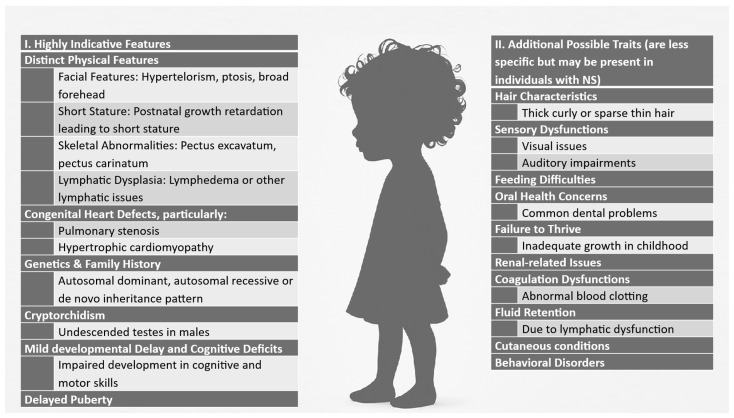
Features of Noonan syndrome. The left panel highlights key features such as distinctive facial morphology, skeletal abnormalities, lymphatic dysplasia, and cognitive impairments, while the right panel details additional possible traits including hair abnormalities, sensory deficits, coagulation issues, and behavioral disorders (adapted after [3,7,10]).

RASopathies, one of the largest categories of diseases characterized by multiple congenital anomalies, encompass a collection of conditions arising from alterations in genes that codify the Ras/mitogen-activated protein kinase (RAS/MAPK) pathway, which regulates cell growth, development, and differentiation [1,2]. These syndromes, such as Noonan syndrome (NS), Cardiofaciocutaneous syndrome, Costello syndrome, Legius syndrome, Neurofibromatosis type 1 and Noonan-like syndrome with loose anagen hair, along with capillary malformation/arteriovenous malformation (CMA/AVM) syndrome exhibit similar clinical features due to disruptions in RAS/MAPK signaling. NS is genetically heterogeneous—several different genes can cause the condition, all of which are involved in the RAS–MAPK signaling pathway, and genetic testing is essential to identify the underlying variants and inform clinical care strategies [3,4]. However, the connections between specific genetic mutations and their associated clinical manifestations are not yet fully understood [4]. Noonan syndrome, OMIM #163950, is a well-known genetic condition characterized by a range of clinical features, and is one of the most common syndromic causes of congenital heart disease [5]. The syndrome has a prevalence of 1 in 1000 to 2500 live births marked by distinctive facial features, developmental delay, and various physical anomalies [3]. NS is typically inherited in an autosomal dominant manner with marked phenotypic variability, but autosomal recessive inheritance (rare cases) was also described [6]. However, most of the cases appear to be sporadic, occurring in individuals with no prior family history of the disorder [3,5]. The phenotype ranges from oligosymptomatic adults with few medical concerns to severely affected newborns with life-threatening heart conditions [3]. NS is characterized by distinctive craniofacial features, short stature, congenital heart defects, and developmental delays. Figure 1 provides a comprehensive overview of the key clinical manifestations of NS [3,7,8].

While clinical features are central to diagnosis, genetic testing can confirm NS in approximately 75% of cases [9].

The NS causal genes from the Ras/MAPK pathway comprise all currently known pathogenic variants (heterozygous) in *BRAF*, *KRAS*, *MAP2K1*, *MRAS*, *NRAS*, *PTPN11*, *RAF1*, *RASA2*, *RIT1*, *RRAS2*, *SOS1*, or *SOS2* genes; in addition, *LZTR1* contributes to the condition through biallelic pathogenic variants [7,10,11]. The *PTPN11* gene variant c.922A>G (p.Asn308Asp) substitution is one of the most prevalent pathogenic variants described to date in NS, occurs mostly as a *de novo* event, but has also been documented to segregate with disease in affected families. Functional studies show that this exon 8 variant in the PTPN11 phosphatase domain mildly increases catalytic activity, supporting its pathogenic role and hotspot status in NS [12].

Effective NS management depend on early diagnosis, and requires multidisciplinary evaluation by pediatricians, cardiologists, endocrinologists, and other specialists to address its complex, varied clinical features [10,13,14].

Overall, NS is a complex genetic condition that can present with a variety of symptoms, a combination of clinical characteristics must be identified to diagnose NS, and in many cases, genetic testing confirms the diagnostic.

The aim of this study was to evaluate a group of patients meeting the clinical criteria for NS (adapted from Van der Burgt) in order to characterize their key clinical features and assess the prevalence of one of the most common pathogenic variants, *PTPN11* c.922A>G, associated with NS. Despite increasing awareness and improved diagnostic tools, data on affected individuals from Eastern Europe remain scarce. To our knowledge, this is the first study describing a cohort of patients with a clinical suspicion of NS in Romania, aiming to provide insights into their evaluation.

## 2. Materials and Methods

### 2.1. Patients

We conducted a retrospective study on patients evaluated into the Children’s Emergency Clinical Hospital in Cluj-Napoca, Romania. Clinical data were collected from 51 patients evaluated between 2018 and 2024 in the Pediatrics and Genetics Departments, patients with clinical suspicion of NS. Data were collected on anamnesis (growth and development, family history), auxologic data, dysmorphic features, cardiac and other internal malformations (ultrasound examination), intellectual/development quotient and other neurologic/psychiatric features, other clinical or paraclinical features (bio-chemical, hormonal, hematological/coagulation, immunological) relevant for this diagnosis. Of these patients, 31 were selected as fulfilling the clinical criteria adapted from Van der Burgt [15]:

1. A typical facial appearance together with at least one additional major criterion or two minor criteria, or
2. Suggestive facial dysmorphology plus two major or three minor signs.

The major criteria are cardiac defects (pulmonary valve stenosis, hypertrophic cardiomyopathy, and/or typical ECG), short stature (under percentile 3), chest wall deformities (pectus carinatum/excavatum), and family history of definite NS or other features (intellectual disability, cryptorchidism, and lymphatic dysplasia). The minor criteria are other cardiac defects, short stature (under percentile 10), broad thorax, first-degree relative(s) with suggestive NS or other features (intellectual disability, cryptorchidism, or lymphatic dysplasia).

Patients clinically suspected of having Noonan syndrome, but who did not fully meet the diagnostic criteria described above, were excluded from this study. The selected patients from the cohort were tested for the pathogenic variant *PTPN11* c.922A>G.

Ethical approval for this study was obtained from the Ethics Committee of the “Iuliu Hațieganu” University of Medicine and Pharmacy from Cluj-Napoca and Children’s Emergency Hospital, Cluj-Napoca, Romania. A signed written informed consent was obtained from the legal guardian for all the subjects included into the study.

### 2.2. DNA Extraction and PCR-RFLP Analyses

Peripheral venous blood samples were collected on EDTA tubes from all patients for DNA extraction. The Wizard Genomic DNA Purification Kit (Promega, Madison, WI, USA) was used, following the manufacturer’s instructions. One patient of our cohort was analyzed by Multiplex Ligation-dependent Probe Amplification (MLPA) using P245 microdeletions probes.

For the Polymerase Chain Reaction–Restriction Fragment Length Polymorphism (PCR-RFLP) procedure, the PCR reaction mixture (PCR Master Mix Thermo Fisher Scientific Inc.,MA, USA) was used; the reaction also included the genomic DNA and specific forward and reverse primers, forward: 5′-GAC ATC AGG CAG TGT TCA CGT TAC-3′ and reverse: 5′-CCT TAA AGT TAC TTT CAG GAC ATG-3′, according to Tartaglia et al. [16].

## 3. Results

Our cohort includes 31 patients consisting of 22 males and 9 females. The median age at evaluation was 6 years (min 11 months; max 17 years and 11 months).

### 3.1. Clinical Characteristics

**Figure 2:**
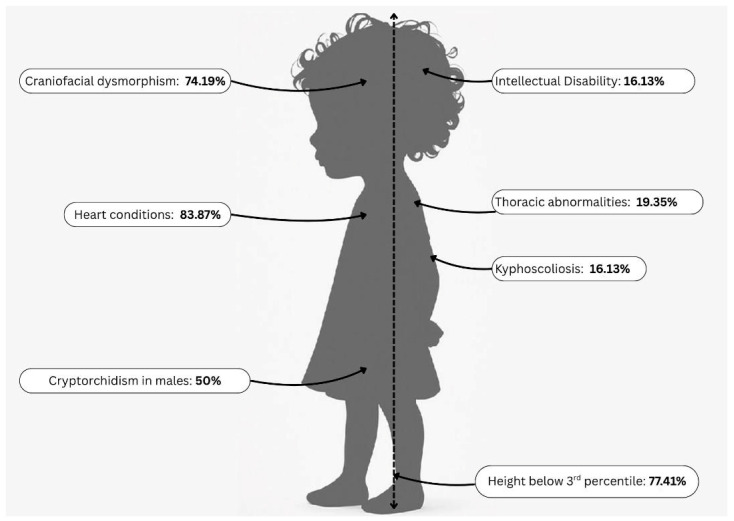
A schematic illustration to better visualize the reported features in our cohort.

Clinical characteristics of the patients with a clinical suspicion of NS are summarized in Table 1 below, showing that in this pilot study cohort, short stature was common, with 77.41% of patients having a height below the 3rd percentile. Most patients had congenital heart defects (detailed in Table 2), and craniofacial dysmorphism was observed in 76.66%, most frequently downslanting palpebral fissures (46.66%) and low-set ears (43.33%). Cryptorchidism was present in 50% of male patients (Figure 2).

The first, a 3-month-old infant, showed predominantly right ventricular hypertrophy with preserved myocardial contractility. The second, an 8-month-old, had non-obstructive hypertrophic cardiomyopathy, and the third patient, assessed at 3.5 years old, had been diagnosed with obstructive hypertrophic cardiomyopathy at 2 years of age. Overall, pulmonary stenosis was present in 61.29% of the patients. Of 19 patients with pulmonary stenosis, 5 had valve dysplasia; 4 of these had both valvular and supravalvular stenosis, while one had isolated pulmonary valve dysplasia.

One patient of our cohort was later diagnosed with WBS by MLPA using P245 microdeletion probes. Another patient was tested using comparative genomic hybridization (CGH) array, revealing 17q.11.2 deletion encompassing NF1 gene, corresponding to Neurofibromatosis 1 microdeletion syndrome.

### 3.2. Genetic Results

Genetic analyses using PCR-RFLP revealed the presence of pathogenic variant *PTPN11* c.922A>G in two of our subjects (representing 6.45%), both with a hypostature below percentile 3 and typical dysmorphism.

This reaction generated 350 bp amplicons. The variant c.922A>G in exon 8 creates a restriction site for the enzyme the *EcoRV* (GAT/ATC) [16]. After digestion with *EcoRV*, the fragments were analyzed through electrophoresis on 2,5% agarose gel, and then examined using a UV transilluminator; the images were captured and evaluated for fragment patterns. The mutated alleles produced two DNA fragments of 246 bp and 104 bp, respectively, while the wild-type allele remained undigested (see Supplementary Figure S1).

### 3.3. Details for Case No. 1 Positive for PTPN11 c.922A>G

This patient was an 11-month-old boy with typical facial dysmorphism: triangular face, downslanting palpebral fissures, hypertelorism, a broad nasal base, low-set ears, a right floating testicle, short stature (SDS for height was −3.67, below the 1st percentile). The echocardiographic examination showed severe supravalvular and pulmonary valve stenosis (see Figures S2 and S3 in the Supplementary Materials).

### 3.4. Details for Case No. 2 Positive for PTPN11 c.922A>G

The patient was a boy with a clinical suspicion of NS based on clinical presentation at 7 years old, but genetic testing was available 5 years later. At the age of 7, he presented facial dysmorphism (hypertelorism, low-set ears, ogival palate), pterygium colli, pectus carinatum, and short stature (SDS for height was −2.75), and growth hormone therapy was initiated. He also had minimal cardiac involvement, including minor aortic insufficiency and minor mitral insufficiency.

## 4. Discussion

Noonan syndrome is a phenotypically and genetically diverse disorder caused by variants in several genes involved in RAS signaling. Due to this genetic variability, testing for NS genes is often complex and involves multiple steps.

We analyzed 31 patients with clinical suspicion of Noonan syndrome and identified two individuals carrying the pathogenic *PTPN11* c.922A>G variant. Both positive patients presented facial dysmorphism (hypertelorism, low-set ears), short stature, and at least one additional clinical sign, fulfilling the Van der Burgt criteria [15]. Diagnosis is considered if a typical facial appearance (major facial criterion) is present and at least one other major criterion (from cardiac defects, short stature below 3rd percentile, chest wall deformities, family history of definite NS, or other major features), or suggestive facial appearance (minor facial criterion) is present, plus either two major criteria or three minor criteria.

Case 1 presented with the most common cardiac abnormality, pulmonary stenosis, while case 2 showed pectus carinatum but only minimal cardiac signs. These two cases illustrate the clinical heterogeneity of the disease.

There is a lack of comprehensive epidemiological data and mutation detection rates; genes associated with Noonan syndrome include *PTPN11* (found in approximately half of NS patients); besides the known genes (*BRAF*, *KRAS*, *MAP2K1*, *MRAS*, *NRAS*, *PTPN11*, *RAF1*, *RASA2*, *RIT1*, *RRAS2*, *SOS1*, *SOS2*, *LZTR1*), several additional genes have been identified in fewer than ten individuals, each associated with a Noonan-syndrome-like phenotype [3,17]. The causative variants are still unidentified in 10–20% of patients, with *de novo* variants being responsible for the majority of NS cases [10]. The association of dysmorphic features such as a triangular face, low-set ears, and hypertelorism, combined with additional diagnostic criteria like short stature, thoracic deformities, or heart problems raises suspicion of NS.

In terms of clinical characteristics, cardiac abnormalities were the most commonly observed feature. Among the cardiac conditions, pulmonary stenosis was the most prevalent, identified in 61.12% of the cohort (see Table 2); this was the most frequently reported cardiac abnormality in most studies (~60%) [4,8,18,19], consistent with the findings of our cohort. Previous research identified hypertrophic cardiomyopathy (HCM) as the second most frequent cardiac lesion in Noonan syndrome, following pulmonary stenosis [4,20]. In our cohort, three patients presented with both PVS and HCM, a combination that was been previously described in Noonan syndrome [20]. Further genetic testing could be valuable in exploring potential associations between specific pathogenic variants and this complex phenotype.

The second most frequent clinical characteristic observed in our cohort was short stature, identified in 24 out of 31 patients (77.41%). In a study published in 2024, short stature was identified in 62.5% of NS cases, with an additional 21.8% having heights between the 3rd and 10th percentile [4]. However, another study of 107 NS patients with *PTPN11* variants reported that only 40% had short stature (height < 3rd percentile for age). The authors suggested that this lower percentage might be because most patients were young children who had not yet completed pubertal growth [21].

The most common combination of features was short stature and pulmonary stenosis, present in 15 patients, approximately half of the cohort (48,38%). Among these patients, five exhibited typical facial dysmorphism, six showed suggestive facial dysmorphism, and in four cases, no facial dysmorphism was reported. Although facial dysmorphism is a key diagnostic feature of Noonan syndrome, its assessment remains subjective, which may account for the lower overall prevalence of 76.66% observed in our cohort. In contrast, the study by Yılmaz Uzman et al. (2024) reported a prevalence of 96% for hypertelorism and 90% for downslanting palpebral fissures, both higher than the rates observed in our patients [4].

In our cohort, cryptorchidism was observed in 50% of cases, a finding comparable to other studies where this feature was present in 44% of patients [21]. Out of the 22 male patients, eight presented a combination of pulmonary stenosis, craniofacial dysmorphism (six typical and two suggestive), and cryptorchidism. Among them, only five also had short stature, with height below the 10th percentile. In some cases, cryptorchidism in males, when associated with other specific features of Noonan syndrome (such as cardiac involvement and facial dysmorphism), could serve as a clinical red flag for suspicion of NS.

Due to overlapping features such as short stature, cardiac defects like pulmonary stenosis, and certain skeletal abnormalities (e.g., pectus excavatum and kyphoscoliosis), the syndromes described further can be easily confused with NS, particularly during adolescence and adulthood, when craniofacial dysmorphism becomes less pronounced.

Table 3 highlights both the similarities that may lead to diagnostic confusion and the distinctive features that facilitate differentiation between these syndromes.

In our case, the boy was diagnosed at 2 months of age. The craniofacial dysmorphism was not suggestive for WBS and also was difficult to assess at that age his psychological traits. For NF1 microdeletion syndrome the hallmark is café-au-lait spots, freckling, neurofibromas, that were not present in our case. In the literature, we found a case report of a patient carrying a 1.69 Mb *de novo* deletion at 17q11.2 adjacent to NF1 gene, who presented with developmental delay, short stature and dysmorphic features (hypertelorism, flat nasal bridge, and posteriorly rotated and low-set ears), and also with no skin modifications like café-au-lait spots were noted [31].

In 2018, when our initial patients were tested, the *PTPN11* c.922A>G variant was chosen for genetic analysis due to the relative simplicity of the testing protocol published by Tartaglia et al. in 2002 and, because this variant accounted for 25% of all *PTPN11* variants, as reported by Romano et al. (2010) [10,16]. Other studies had found the same variant in different percentages varying from 0 to 18,25% (see Table 4). The variation in the reported prevalence of this variant across studies may be attributed to differences in patient selection criteria. For instance, Bertola et al. (2006) utilized established clinical diagnostic criteria, such as those proposed by Van der Burgt et al., for patients’ inclusion [15,32]. In contrast, Sznajer et al. (2007) employed a systematic enrollment approach based on the chronological order of sample submission for *PTPN11* molecular testing, and reported a higher prevalence of this variant [33]; see Table 4. Additionally, population-specific genetic variation may also contribute to differences in observed frequencies.

The low incidence rate observed in our study on Romanian patients with NS can be explained by the fact that we analyzed a smaller cohort, which may have limited the detection of the specific variant (*PTPN11* c.922A>G) compared to larger studies. Additionally, the lack of detailed clinical documentation in some cases—for instance, the absence of a thorough description of craniofacial dysmorphism in seven patients —may have contributed to diagnostic over-inclusion or misclassification. Accurate recognition of characteristic facial features is essential in NS [40]; yet, these features often evolve and become more subtle with age, further complicating diagnosis [41]. The lack of detailed clinical documentation in some cases—for instance, absence of a thorough description of craniofacial dysmorphism in several patients—may have contributed to diagnostic over-inclusion or misclassification.

This discrepancy might also be attributed to the genetic characteristics and variability of the Romanian population, which differ from those of other populations studied in the literature. Genetic diversity and population-specific factors likely play a role in the observed differences.

Notably, external hydrocephalus has been reported in association with *PTPN11* variants, including a fetal case carrying the c.923A>G substitution, which is adjacent to the c.922A>G variant identified in our cohort. There are reports describing patients with the same genotype or amino acid substitution who presented with Arnold–Chiari malformation or external hydrocephalus [42,43]; however, this feature was not observed in our patients. These observations suggest that variants within this mutational hotspot may contribute to neurodevelopmental anomalies, underscoring the importance of detailed neuroimaging evaluation in individuals with NS carrying *PTPN11* mutations. An important limitation of our study is that genetic testing was restricted to the *PTPN11* c.922A>G variant, and therefore, the genotypes of the remaining patients could not be determined. Broader molecular analyses would offer a more complete view of the underlying genetic landscape. Further studies involving larger Romanian cohorts are needed to better characterize the pathogenic variants prevalent in this population. One path for future research could be Sanger sequencing of exons 3, 7, 8, 12, and 13, which harbor the most pathogenic variants of *PTPN11* gene. Additionally, the application of advanced genetic technologies, such as next-generation sequencing (NGS) targeting, to all genes involved in the RAS pathway, represents a promising direction for future research.

Other studies on Romanian patients have underscored the highlight the heterogeneous clinical spectrum and management challenges of NS. Făgărășanu et al. (2022) emphasized the prognostic implications of *RAF1* variants, particularly in a patient with hypertrophic cardiomyopathy and arrhythmic risk [44]. Similarly, Harpa et al. demonstrated how minimally invasive techniques can be successfully adapted for complex cardiovascular phenotypes, such as mitral valve pathology associated with thoracic deformities [45]. Additionally, a case of an NS young patient operated for pulmonary stenosis and septal atrial defect was diagnosed with juvenile onset of ankylosing spondylitis and Crohn’s disease have been documented in a Romanian patient by Nicola et al. in 2019 [46]. In addition, in 2021, Radu et al. reported a NF1–Noonan overlap syndrome with pulmonary stenosis and hypertrophic cardiomyopathy, underscoring the genetic and clinical variability in RASopathies [47].

The lower incidence of the *PTPN11* c.922A>G variant in our Romanian cohort may reflect not only the limited sample size but also possible population-specific genetic variability. Differences in allelic distribution and mutational hotspots across populations have been reported in other RASopathies, suggesting that genetic background may influence variant prevalence. Broader molecular screening using NGS could clarify whether these findings represent true population-related variation or reflect sampling limitations.

To the best of our knowledge, this is the first report on a Romanian cohort of patients with Noonan syndrome, modestly yet meaningfully contributing novel data to the international literature and supporting the need for further multicenter studies. These findings may help refine genotype–phenotype correlations and support earlier diagnosis, individualized surveillance, and multidisciplinary management strategies.

## 5. Conclusions

This study presents data from a pilot investigation into the clinical and partial genetic characteristics of NS in Transylvania, with a Romanian cohort. The findings reaffirm the phenotypic variability of NS, with pulmonary stenosis, as the most prevalent cardiac manifestation. Craniofacial dysmorphism and short stature were also frequently observed, which is consistent with previous reports. Genetic analysis identified the pathogenic *PTPN11* c.922A>G (p.Asn308Asp) variant in 6.45% of the studied patients, a lower frequency compared to other cohorts (11 studies). This result suggests possible genetic heterogeneity within the Romanian population, highlighting the importance of broadening genetic testing beyond *PTPN11* to other genes in the RAS/MAPK pathway. The presence of additional syndromes, such as WBS and NF1 microdeletion syndrome, within our cohort further underscores the need for comprehensive genetic evaluation to ensure accurate diagnosis and management. Additionally, we underscore the importance of expanding research to include whole-exome or whole-genome sequencing to uncover novel variants associated with NS. This study contributes valuable epidemiological data and genotype–phenotype correlation information for NS in the Romanian population, facilitating improved diagnostic, support personalized management, and counseling strategies.

## Figures and Tables

**Table 1:** Clinical features observed in patients with a clinical suspicion of NS in the study cohort (auxological parameters, dysmorphic features, congenital heart defects, skeletal abnormalities, and other associated traits).

Key Clinical Features in the Study Cohort	Number of Patients, *n* (%)
Height below third percentile	24 (77.41%)
Height between percentile 3 and 10	1 (3.22%)
Heart conditions	26 (83.87%)
Craniofacial dysmorphism	23 (74.19%)
Triangular face	5 (16.66%)
Hypertelorism	10 (33.33%)
Palpebral ptosis	2 (6.63%)
Downslanting palpebral fissures	14 (46.66%)
Low set ears	13 (43.33%)
Pterygium colli	3 (9.67%)
Thoracic abnormalities	6 (19.35%)
Broad thorax	1 (3.33%)
Pectus carinatum	2 (6.45%)
Pectus excavatum	3 (9.67%)
Wide-spaced nipples	5 (16.13%)
Kyphoscoliosis	5 (16.13%)
Intellectual disability	5 (16.13%)
Cryptorchidism in males	11 (50%)

**Table 2:** Distribution of various congenital heart defects among the study cohort.

Congenital Heart Defect	Number of Patients, *n* (%)
PVS	16 (51.61%)
PVS and HCM *	3 (9.68%)
Pulmonary valve dysplasia	1 (3.23%)
Left HCM	1 (3.23%)
Atrial sept defect operated	1 (3.23%)
Atrial septal aneurysm with left–right shunt	1 (3.23%)
Small muscular sept defect	1 (3.23%)
Patent foramen ovale	2 (6.45%)
No heart condition	5 (16.13%)

HCM: hypertrophic cardiomyopathy; PVS: pulmonary valve stenosis. * Three patients were diagnosed with both pulmonary stenosis and hypertrophic cardiomyopathy.

**Table 3:** Overlapping and distinctive features of Williams–Beuren syndrome, Neurofibromatosis 1 microdeletion syndrome compared to Noonan syndrome.

Features	Noonan Syndrome NF1	Williams–Beuren Syndrome	Neurofibromatosis Type 1 Microdeletion Syndrome	References
Genetic Causes	Variants in *BRAF*, *KRAS*, *MAP2K1*, *MRAS*, *NRAS*, *PTPN11*, *RAF1*, *RASA2*, *RIT1*, *RRAS2*, *SOS1*, *SOS2*, *LZTR1* (RAS/MAPK pathway); several additional genes, each linked to an NS-like phenotype, were identified in fewer than ten individuals	Hemizygous deletion of 1.5 to 1.8 Mb, approx. 28 genes from 7q11.23; is a contiguous gene deletion syndrome	Deletion of 17q11.2 (including NF1 gene)	[3,17,22,23,24]
Alternative name	- Male Turner syndrome - Female pseudo-Turner syndrome - Turner phenotype with normal karyotype	- Williams syndrome - Deletion 7q11.23 syndrome - Monosomy 7q11.23	- Neurofibromatosis–Noonan Syndrome (NFNS) - Del(17)(q11) - Monosomy 17q11 - NF1 microdeletion syndrome	[24]
Gene/Locus MIM number	*176876	*130160	*613113	[24]
Phenotype MIM number	#163950	#194050	#601321	[24]
Inheritance pattern	Mostly *de novo* or AD variants; AR forms of NS include NS2, caused by variants in the *LZTR1* gene, and *NS14*, caused by variants in the *SPRED2* gene	Mostly *de novo*, AD	Mostly *de novo* but can be inherited AD	[6,25]
Facial features	Triangular face, hypertelorism, ptosis, low-set ears: similar facial phenotypes to WBS modulated by ethnic background	“Elfin-like” face, broad forehead, full cheeks, wide mouth	Coarse facial features, hypertelorism, down-slanting palpebral fissures	[22,23,26,27]
Growth and stature	Short stature, postnatal growth retardation	Short stature, failure to thrive in infancy	Short stature, variable growth delay	[21,27]
Cardiac defects	In up to 90% of patients, PVS, HCM	Supravalvular aortic stenosis, pulmonary artery stenosis	Pulmonic stenosis, other congenital heart defects	[3,28]
Cognitive and developmental delay	Mild-to-moderate intellectual disability, learning difficulties	Intellectual disability, friendly/social personality	Developmental delay, speech delay, learning disabilities	[3,21,29]
Skeletal abnormalities	Pectus excavatum, pectus carinatum, scoliosis	Joint laxity, skeletal abnormalities	Pectus abnormalities, scoliosis	[3,21]
Skin manifestations	Generally normal	Soft skin, premature aging of the skin	Café-au-lait spots, axillary/inguinal freckling, multiple neurofibromas	[3]
Behavioral features	Varied; some social difficulties, verbal communication challenges	Overly friendly, anxiety, attention deficits, fear of loud noises	Attention deficits, possible ASD-like traits	[3,10]
Renal and urinary abnormalities	Sometimes present, cryptorchidism in males	Common, including renal artery stenosis	Genitourinary abnormalities, cryptorchidism in males; increased risk of HT	[3]
Cancer risk	Slightly increased risk of certain malignancies (e.g., leukemia, neuroblastoma)	No significant cancer predisposition	Increased risk of malignancies (due to *NF1* gene deletion); e.g., plexiform neurofibromas, optic gliomas, malignant peripheral nerve sheath tumors	[3,10]
Sensory conditions vision issues, hearing changes	Strabismus, refractive errors, sensorineural hearing loss	Hyperacusis (sensitivity to sound), strabismus	Optic pathway gliomas, Lisch nodules (iris hamartomas), vision impairment	[3]
Distinctive features	Wide-spaced nipples, short/webbed neck, coagulation abnormalities	Outgoing personality, musical affinity, strong verbal skills	Café-au-lait macules, neurofibromas, and an increased risk of tumors	[3,10]
Diagnostic clues	Cardiac defects + short stature + facial dysmorphism	Supravalvular aortic stenosis + hypersociability + “elfin-like” face	Café-au-lait spots + neurofibromas	[3,26]
Prevalence	1–5/10,000	approx. 1/7500	Not known; about 5% of NF1 cases are reported to have deletions of the entire NF1 gene	[30]

Legend: AD—autosomal dominant; AR—autosomal recessive; ASD—autism spectrum disorder; HCM—hypertrophic cardiomyopathy; HT—hypertension; MIM—Mendelian Inheritance in Man; NS—Noonan syndrome; PVS—pulmonary valve stenosis; WBS—Williams–Beuren syndrome. An asterisk (*) before a MIM number indicates a gene entry, while a number sign (#) before a MIM number indicates a phenotype entry.

**Table 4:** Comparison of *PTPN11* variants in different studies.

Study [Reference]	Total Number of Patients	Number of PTPN11 Variants	Percentage of PTPN11 Variants	Number of PTPN11 C.922A>G Variants	Percentage of PTPN11 C.922A>G Variants
Orlova et al., 2024 [12]	456	107	23.46	23	5.04
Sznajer et al., 2007 [33]	272	104	38.24	19	18.2
Tartaglia et al., 2002 [16]	112	54	48.21	17	15.18
Brasil et al., 2010 [34]	95	42	44.21	11	11.58
Zepeda-Olmos et al., 2024 [35]	91	43	47.25	7	7.69
Bertola et al., 2006 [32]	50	21	42.00	0	0.00
Yoshida et al., 2004 [36]	45	18	40.00	2	4.44
Ferreira et al., 2007 [37]	33	16	48.48	5	15.15
Kosaki et al., 2002 [38]	21	7	33.33	1	4.76
Athota et al., 2020 [21]	363	107	29.47	12	3.30
Ouboukss et al., 2024 [39]	61	17	41.4%	10	16.39
Our study	31	Not known	Not known	2	6.45

## Data Availability

The original contributions presented in this study are included in the article and Supplementary Materials. Further inquiries can be directed to the corresponding author.

## Supplementary Materials

The following supporting information can be downloaded at https://www.mdpi.com/article/10.3390/diagnostics15212753/s1, Figure S1: Agarose gel electrophoresis for PCR-RFLP for *PTPN11*, c.922A>G mutation; Figure S2:Echocardiography, parasternal short axis view, CW Doppler, severe pulmonary valve stenosis; Figure S3: Echocardiography, parasternal short axis view, Color Doppler, severe supravalvular and pulmonary valve stenosis.

## Abbreviations

The following abbreviations are used in this manuscript:

AD	Autosomal dominant
AR	Autosomal recessive
ASD	Autism spectrum disorder
CGH	Comparative genomic hybridization
CMA/AVM	Capillary malformation/arteriovenous malformation
ECG	Electrocardiogram
HCM	Hypertrophic cardiomyopathy
HT	Hypertension
MIM/ OMIM	Mendelian Inheritance in Man/ Online Mendelian Inheritance in Man
MLPA	Multiplex Ligation-dependent Probe Amplification
NGS	Next-generation sequencing
NS	Noonan syndrome
PCR-RFLP	Polymerase Chain Reaction–Restriction Fragment Length Polymorphism
PTPN11	Protein Tyrosine Phosphatase, Non-Receptor Type 11
PVS	Pulmonary valve stenosis
RAS/MAPK	Rat Sarcoma/Mitogen-Activated Protein Kinase
SDS	Standard Deviation Score
WBS	Williams–Beuren syndrome
