# Supplementary material for: Clinical and Genetic Characterization of Noonan Syndrome in a Romanian Cohort from Transylvania: Details on PTPN11 c.922A>G Variant and Phenotypic Spectrum
Source: Diagnostics (Basel). 2025 Oct 30;15(21):2753. doi: 10.3390/diagnostics15212753 (PMC12607955; doi:10.3390/diagnostics15212753)
Supplement: Supplementary file 1 [file diagnostics-15-02753-s001.zip › diagnostics-3868228-supplementary.pdf]

Supplementary Materials

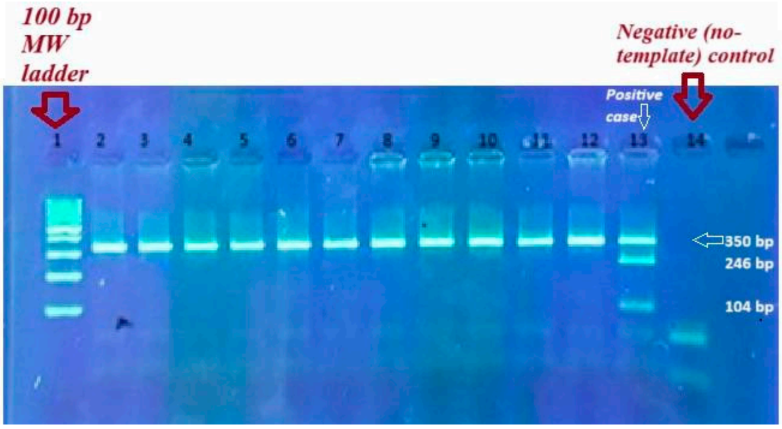

Figure S1. Agarose gel electrophoresis for PCR-RFLP for *PTPN11*, c.922A>G mutation.

Agarose gel 2,5% was used to separate the fragments resulted after digestion of PCR products with *EcoRV* restriction enzyme; these fragments were visualized under a UV transilluminator. Lane 1: 100 bp molecular weight ladder; Lane 2-12: samples negative for *PTPN11*, c.922A>G mutation, showing a single band at 350 bp; Lane 13 – positive case showing 2 fragments of 246 bp and 104 bp, obtained after restriction enzyme digestion. Lane 14: negative (no-template) control.

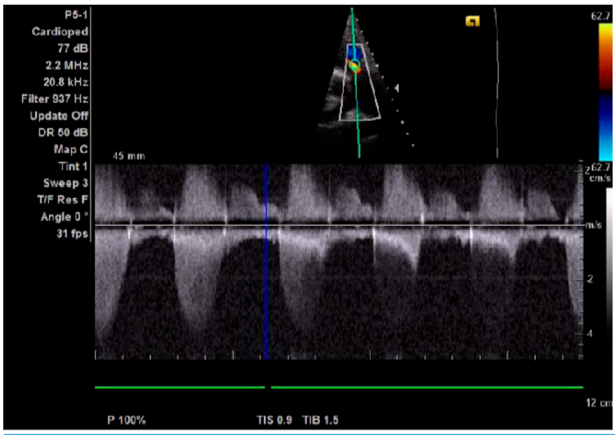

Figure S2. Echocardiography, parasternal short axis view, CW Doppler, severe pulmonary valve stenosis.

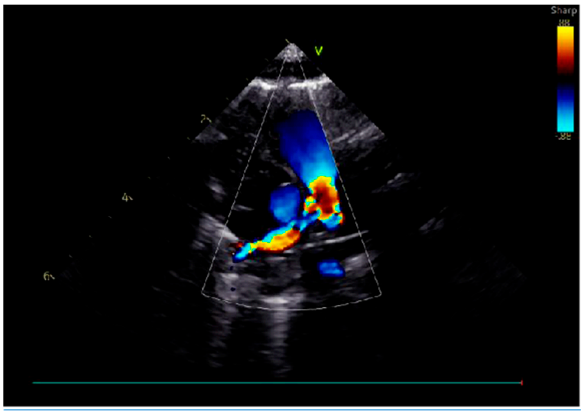

Figure S3. Echocardiography, parasternal short axis view, Color Doppler, severe supravalvular and pulmonary valve stenosis.
